# The anatomy of the human medial forebrain bundle: Ventral tegmental area connections to reward-associated subcortical and frontal lobe regions

**DOI:** 10.1016/j.nicl.2018.03.019

**Published:** 2018-03-18

**Authors:** Volker Arnd Coenen, Lena Valerie Schumacher, Christoph Kaller, Thomas Eduard Schlaepfer, Peter Christoph Reinacher, Karl Egger, Horst Urbach, Marco Reisert

**Affiliations:** a Department of Stereotactic and Functional Neurosurgery, Medical Center, Freiburg University, Germany; b Department of Neuroradiology, Medical Center, Freiburg University, Germany; c Department of Interventional Biological Psychiatry, Medical Center, Freiburg University, Germany; d Department of Medical Physics, Medical Center, Freiburg University, Germany; e Medical Faculty, Freiburg University, Germany; f Department of Neurology, Medical Center, Freiburg University, Germany; g Medical Psychology and Medical Sociology, Faculty of Medicine, University of Freiburg, Germany

**Keywords:** Brain, Deep brain stimulation, Depression, Human, Medial forebrain bundle, Normal anatomy, Obsessive compulsive disorder, TMS

## Abstract

**Introduction:**

Despite their importance in reward, motivation, and learning there is only sparse anatomical knowledge about the human medial forebrain bundle (MFB) and the connectivity of the ventral tegmental area (VTA). A thorough anatomical and microstructural description of the reward related PFC/OFC regions and their connection to the VTA - the superolateral branch of the MFB (slMFB) - is however mandatory to enable an interpretation of distinct therapeutic effects from different interventional treatment modalities in neuropsychiatric disorders (DBS, TMS etc.). This work aims at a normative description of the human MFB (and more detailed the slMFB) anatomy with respect to distant prefrontal connections and microstructural features.

**Methods and material:**

Healthy subjects (*n* = 55; mean age ± SD, 40 ± 10 years; 32 females) underwent high resolution anatomical magnetic resonance imaging including diffusion tensor imaging. Connectivity of the VTA and the resulting slMFB were investigated on the group level using a global tractography approach. The Desikan/Killiany parceling (8 segments) of the prefrontal cortex was used to describe sub-segments of the MFB. A qualitative overlap with Brodmann areas was additionally described. Additionally, a pure visual analysis was performed comparing local and global tracking approaches for their ability to fully visualize the slMFB.

**Results:**

The MFB could be robustly described both in the present sample as well as in additional control analyses in data from the human connectome project. Most VTA- connections reached the superior frontal gyrus, the middel frontal gyrus and the lateral orbitofrontal region corresponding to Brodmann areas 10, 9, 8, 11, and 11m. The projections to these regions comprised 97% (right) and 98% (left) of the total relative fiber counts of the slMFB.

**Discussion:**

The anatomical description of the human MFB shows far reaching connectivity of VTA to reward-related subcortical and cortical prefrontal regions - but not to emotion-related regions on the medial cortical surface - realized via the superolateral branch of the MFB. Local tractography approaches appear to be inferior in showing these far-reaching projections. Since these local approaches are typically used for surgical targeting of DBS procedures, the here established detailed map might - as a normative template - guide future efforts to target deep brain stimulation of the slMFB in depression and other disorders related to dysfunction of reward and reward-associated learning.

## Highlights

• The MFB is robustly described with advanced DTI methods.
• Connections of VTA reach cortical and subcortical reward related regions.
• Parcellation into 8 segments are shown in a normative cohort (*n* = 55).
• Normative anatomy will help guide therapeutic interventions.

We dedicate this work to Jaak Panksepp, Ph.D. (*1943 - †2017).

## 1 Introduction

The medial forebrain bundle (MFB) is an important structure for reward and motivation in the mammalian brain (Wise, 2005; Olds & Fobes, 1981; Zacharopoulos et al., 2016; Morgane & Panksepp, 1981; Panksepp, 1985; Alcaro et al., 2007) that also plays a central role in human affective disorders (e.g. depression, obsessive compulsive disorder) (Russo & Nestler, 2013; Döbrössy et al., 2015; Schlaepfer & Lieb, 2005) particularly for disease regulation (Coenen et al., 2011). The anatomy of the human medial forebrain bundle (MFB) was initially described in vivo in the context of psychotropic effects of deep brain stimulation (DBS) using diffusion tensor imaging and fiber tracking (Coenen et al., 2009). A detailed anatomical description revealed converging projections to the prefrontal cortex (PFC) shared with the anterior thalamic radiation (ATR) besides the expected subcortical connections to the ventral striatum (nucleus accumbens, NAC) which are reached over the anterior limb of the internal capsule (ALIC) (Coenen et al., 2012a). Similar findings were reported by others in part using different tractographic approaches modalities (Zacharopoulos et al., 2016; Bracht et al., 2014a; Bracht et al., 2014b; Bracht et al., 2015; Gálvez et al., 2014; Anthofer et al., 2015; Hana et al., 2015; Cho et al., 2015; Owens et al., 2016; Fenoy et al., 2016).

The clinical significance of the MFB for the treatment of affective disorders has only recently become evident (Coenen et al., 2011). For instance, Blood et al. found microstructural changes in the subcortical reward pathways of patients with major depression (Blood et al., 2010a). Bracht et al. observed distinct effects on the microstructure in a slMFB sub-segment which were associated with the melancholic depression phenotype. In this context microstructural slMFB changes were also reported with respect to anhedonia (Bracht et al., 2014a; Bracht et al., 2014b).

Furthermore, the superolateral branch of the MFB (slMFB) has been discovered as a target for DBS in treatment resistant depression (Coenen et al., 2011; Schlaepfer et al., 2014), bipolar disorder (Gippert et al., 2016), and obsessive compulsive (Coenen et al., 2016) disorder with encouraging in part long term results in small single center pilot series for depression (Fenoy et al., 2016; Schlaepfer et al., 2013; Bewernick et al., 2017).

However, choosing the slMFB as a possible target site for chronic DBS in depression (Fenoy et al., 2016; Schlaepfer et al., 2013) and OCD (Coenen et al., 2016) demands a more detailed knowledge about MFB anatomy per se and specifically about its projections to the PFC. In fact, DBS of other subcortical target regions (vc/vs = ventral capsule ventral striatum; cg25 = Brodman's area 25) for major depression has been shown to partly exert remote influence on prefrontal regions (Riva-Posse et al., 2017; Riva-Posse et al., 2014; Noecker et al., 2018) and their adjacent white matter (WM) tracts, especially the anterior prefrontal cortex (aPFC, BA10). The therapeutic overlap related to the PFC is interesting for the interpretation of results and therapeutic efficacy with respect to existing distinct disease phenotypes. Moreover, non-invasive therapies like electroconvulsive therapy (ECT) and transcranial magnetic stimulation (TMS) exert their therapeutic effects by possibly affecting very similar prefrontal regions (Cusin & Dougherty, 2012), opening the road for more personalized and focal interventions.

In this respect, possible microstructural differences of distinct slMFB segments in the diseased as opposed to a control population might be of interest in order to understand the antidepressant effects of any intervention in major depression. Bracht et al. have described a tripartite cortical segmentation of the slMFB projecting to lateral and medial orbitofrontal cortex (OFC) and dorsolateral prefrontal cortex (dlPFC) but without a clear anatomical and descriptive focus (Bracht et al., 2014a). Zacharopolus et al. applied dRL-tractography in conjunction with McDESPOT (Multi-Component Driven Equilibrium Single Pulse Observation of T1 and T2) and have looked at the slMFB as one of the hedonic hubs in the human brain (Zacharopoulos et al., 2016).

Although all these findings appear promising and important, the MFB parcellations used in these studies were not anatomically driven. Effective personalized and focal interventions however require detailed knowledge of the individual anatomy and microstructural pathology of fiber connections between reward-related subcortical and cortical structures.

Thus, with the growing interest in the effects of MFB alterations and its direct manipulation through DBS and other non-invasive stimulation technologies, the formal description of its structure in a common atlas space gains importance. To the best of our knowledge, besides an anatomical description that focuses mainly on subcortical structures (12), a detailed description of the anatomy of the MFB – and the slMFB - particularly with respect to PFC's reward associated regions in a normalized atlas space is as of yet not available.

Therefore, here we report the normative reconstruction of the MFB in a larger cohort of 55 normal controls using an advanced and state-of-the-art DTI-based fiber tracking algorithm (Reisert et al., 2011). The main objective was to anatomically depict the far reaching subcortico-cortical connections between the VTA and the PFC/OFC in a common atlas space (MNI 152 (Montreal Neurological Institute,Canada, Brain Template), 6th generation), by that describing detailed normal MFB anatomy. We further investigated microstructural features based on a new sub-segmentation of the slMFB that is derived from parceling the prefrontal cortex derived from the Desikan/Killiany atlas (Desikan et al., 2006) again using the global tracking approach. Note that the structure described and mainly scrutinized here is the superolateral branch of the MFB (slMFB). The concept of inferomedial (imMFB) – representing the phylogenetically older and lateral hypothalamic part, which is not in detail regarded here - and superolateral MFB - as the evolutionary younger and cortex connecting structure - in our eyes is still valid (Coenen et al., 2009; Coenen et al., 2012a).

## 2 Methods

### 2.1 Subjects

Healthy subjects (*n* = 55; mean age ± SD, 40 ± 10 years; 32 females) were recruited. The cohort presented here is part of a project that was reviewed by Freiburg University ethics committee (no. 528/15). Subjects were screened for depressive symptoms with the German version of the Beck's depression inventory (BDI-II; (Beck et al., 1996)) and excluded if their score was higher than 13 points, corresponding to the cutoff value for ‘minimal or no depressivity’; The mean BDI-II score for depressivity was 3.8 (±3.7), none of the subjects showed signs of depression.

### 2.2 Magnetic resonance imaging

Subjects were scanned on a Siemens 3 T TIM PRISMA using an SE EPI sequencewith a TE = 88 ms and TR = 2008 ms, bandwidth 1780 Hz, flip-angle 90°, GRAPPA factor 2, SMS factor 3 with 17 non-diffusion weighted images, 2*58 images with b-factor b = 1000 and 2000 s/mm^2^; with an in-plane voxel size of 1.5 mm × 1.5 mm and a slice thickness of 3 mm. The overall sequence takes about 6 min of scan time. One phase-encoding flipped b = 0 image was acquired, which is used for distortion correction (FSL's top up (Andersson et al., 2003)). Additionally, a T1-weighted structural dataset was acquired, resolution 1 mm isotropic, TR = 2500 ms, TE = 2.82 ms.

### 2.3 Diffusion analysis and fiber tracking in subject space

The diffusion weighted images were first denoised by a post-processing technique which uses random matrix theory (see (Veraart et al., 2016) for details). This is followed by a Gibbs artifact removal (Kellner et al., 2015) based on local sub-voxel shifts. This step is particularly important for all quantitative DTI indices, which are usually highly influenced by the Gibbs artifact. Then, images were corrected for EPI distortions by FSL's top up (Andersson et al., 2003) and finally up-sampled to isotropic resolution by an edge preserving interpolation approach (Reisert et al., 2017). For all subjects the anatomical reference scans were segmented using VBM8 (http://www.fil.ion.ucl.ac.uk/spm). White matter probability maps were thresholded at a probability of 0.5 to determine the area of fiber reconstruction.

For tractography we mainly followed a global approach (Reisert et al., 2011). As opposed to local walker-based tractography, global fiber tracking tries to find a fiber configuration that best explains the acquired diffusion weighted MRI data. Practically, the optimization process is similar to a polymerization process, where initially the streamlines are short and fuzzy, while during optimization connections proliferate and fibers become more and more congruent with the data. The algorithm is based on so called simulated annealing. Initially the system is at a rather high temperature, and the temperature is slowly decreased during iterations to obtain more and more accurate results. Usually global fiber tractography is found to be less sensitive to noise and the fiber density is directly related to the measured data itself. We followed the algorithm proposed in (Reisert et al., 2011). The provided toolbox contains two parameter sets, we have chosen the ‘dense’ preset. Additionally, to increase the reproducibility, we increased the number of fibers by the following accumulation strategy: After the cooling down phase, the temperature is increased again to 0.1 and the state is further iterated for 10^7^ iterations. This procedure is iterated over 5 rounds and the tracts resulting from each round are accumulated to obtain one final tractogram which is 5 times larger than the initial one (this approach is also presented in {Schumacher et al. 2018, under review}). Further, diffusion tensors were estimated (https://www.uniklinik-freiburg.de/mr-en/research-groups/diffperf/fibertools.html). For tensor estimation, the b = 2000 shell was neglected. In addition to diffusion tensor estimation, also diffusion microstructure indices (DMI) based on an 3-compartment model were estimated, according to (Reisert et al., 2017). These comprised volume fractions of the intra- and extra-axonal compartment.

### 2.4 Fiber tracking in group space

An MNI diffusion group template was generated in order to simply demonstrate a prototypical tracking of a single MFB on the group level DTI data since MFB tracking on the single-subject level (not shown) lead to ambiguous and blurry results that did not illustrate the complete slMFB extension with was wanted here. Advantages of the human connectome project (Human Connectome Project (HCP) database (https://ida.loni.usc.edu/login.jsp)). data over our own group template were higher B-value, higher resolution and a larger subject count. For this control analysis, we used data from 80 subjects from the Human Connectome Project (Q1) data corpus (resolution 1.25 mm isotropic, three b-shells with 1000, 2000, 3000, for details see (Glasser et al., 2013). Normalization to MNI space was performed using the provided deformation fields on the raw diffusion weighted data. For the reorientation of the HARDI data the local Jacobian matrix was used. The MNI diffusion template served for representation/visualization of the MFB from the low-noise, high-quality average HCP dataset to show the “prototypical” appearance of the MFB. For selection criteria cf. Section 2.5.

### 2.5 Reconstruction of the slMFB

To select the superolateral medial forebrain bundle from the globally reconstructed connectome of the individual subjects we applied the following fiber selection strategy: First, to be selected, a fiber had to visit the therapeutic triangle of the lateral VTA, which we defined by a spherical region of radius 3 mm with center (±6, −12, −8) in MNI space. This choice of common MNI coordinates is the result of previous multiple tracking efforts with deterministic tractography (Coenen et al., 2012a; Schlaepfer et al., 2013) now applied in the MNI space. Tracking in this triangle between red nucleus, subthalamic nucleus/substania nigra and mammillo-thalamic tract (cf. Fig. 2) had previously shown to be most efficacious in showing the slMFB on the single subject level. Second, the fiber had to connect the frontal and basal regions, where frontal and basal was defined in MNI space by y > 18 and z < −8, respectively. Lastly, the fiber had to be exclusively on either the right or left hemisphere. This selection strategy was applied to all fibers returned from the global tractography algorithm. The MNI reference was obtained from the corresponding VBM8 segmentation. The same strategy was also applied to the two local seed-based tractography approaches by seeding in the above described spherical region and constraining the fiber terminals as described above (cf. Section 2.9).

### 2.6 Decomposition into sub-bundles

**Fig. 1:**
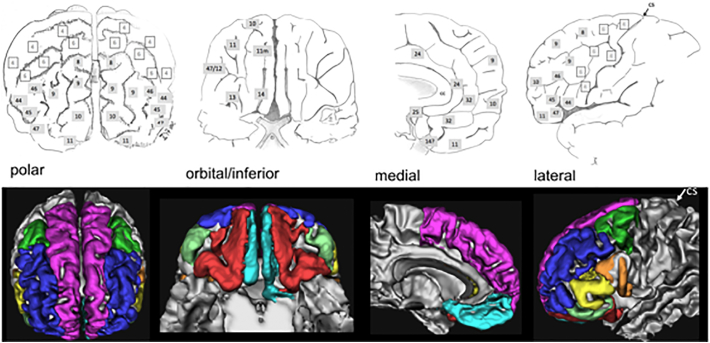
Brodmann areae ((Brodmann, 1909), upper panel. Cortical masks for prefrontal cortex parcellation (Desikan/Killiany (Desikan et al., 2006)) were used in order to identify far-reaching VTA-connections. Cs, central sulcus. Dark grey tags represent PFC related Brodmann regions.

We further subdivided the selected slMFB fiber tracts into eight different sub-bundles by using the Desikan-Killiany atlas (Desikan et al., 2006). The following prefrontal cortical parcels were used (nomenclature in analogy to (Desikan et al., 2006)): lateral orbitofrontal (red), medial orbitofrontal (turquoise), rostral middlefrontal (blue), superior frontal (including frontal pole, pink), pars caudalis (of middle frontal gyrus, green); pars triangularis (yellow), pars opercularis (orange), and pars orbitalis **(**light green) of the inferior frontal gyrus (cf. Fig. 1). Each of these prefrontal segments was taken as an additional selection criterion for the terminals of the selected streamlines from the reconstructed MFB. As the cortical parcellations are rather thin, the terminal projections of our streamlines (the distal 20 mm) were tested for their presumed ending in the respective cortical ROI. Thus, the In Table 1 lists the relative distribution of streamlines reaching the cortical parcels compared to all streamlines obtained using the MFB selection criteria. Around 80% of those reached one of the prefrontal regions. The residual streamlines projected mostly temporally. A smaller and negligible portion was falsely attracted by the anterior commissure.

**Table 1:** Mean values of DTI and DMI parameters were achieved with the global tracking approach (cf. 2.5, 2.6) averaged in the tract defined regions and are reported. Bold segments comprise the majority of the percentage MFB projection. All ADC values are given in μm^2^/s, FA and intra/extra-axonal fractions are given in parts per thousands. Further, percentages of fibers reaching specific subcortical WM targets are given relative to all frontal projections. Note that, the percentages do not sum up to 100%, because the sub-bundles are not fully disjoint due to streamlines at the boundaries of the cortical parcells.

Cortical region (Deskian/Killiany)	FA	axial ADC	mean ADC	radial ADC	extra axonal	intra axonal	% of frontal projection
Right							
**Superiorfrontal**	354	923	799	549	376	451	**38%**
**Rostralmiddlefrontal**	341	921	802	564	386	437	**35%**
**Lateralorbitofrontal**	342	941	821	581	382	433	**24%**
Parstriangularis	365	938	810	557	367	451	15%
Parsorbitalis	362	942	816	565	371	445	11%
Parsopercularis	414	917	776	496	354	479	2%
Medialorbitofrontal	377	942	811	548	374	447	5%
Pars caudalis (of middlefrontal gyrus)	422	908	766	479	347	489	1%

Left							
**Superiorfrontal**	351	939	815	566	379	439	**31%**
**Rostralmiddlefrontal**	342	933	812	571	382	438	**35%**
**Lateralorbitofrontal**	333	953	834	597	381	427	**32%**
Parstriangularis	363	950	823	569	364	449	16%
Parsorbitalis	364	954	827	572	369	443	8%
Parsopercularis	405	949	810	531	348	475	3%
Medialorbitofrontal	356	960	833	580	377	433	7%
Pars caudalis (of middlefrontal gyrus)	421	928	784	496	336	500	2%
Full MFB (r)	322	919	805	577	396	427	28,489 SL
Frontal projections (r)	325	917	802	572	395	430	79% of full
Full MFB (l)	319	927	814	587	397	421	27,393 SL
Frontal projections (l)	322	926	812	583	396	423	83% of full

### 2.7 Comparison to Brodmann parceling of the PFC

In order to facilitate further discussion, resultant sub-segments (Desikan et al., 2006) were compared to respective Brodmann regions (Brodmann, 1909) (cf. Fig. 1).

### 2.8 Fiber-density maps and DTI/DMI indices

For each subject fiber density maps of the full slMFB and of the sub-bundles were rendered at a resolution of 3 × 3 × 3 by trilinear interpolation. Then, the fiber density maps were normalized to group space and thresholded at a value of 1 mm of streamline length per voxel, and group averages of the streamline indicator images were build. In order to understand the true extension of the full slMFB the structure was overlaid onto a T1W template in MNI (axial, coronal, sagittal). To get an impression of the distribution of sub-bundles, relative maps were created by computing for each voxel the percentage of streamlines contained in the sub-bundle compared to all slMFB streamlines.

To get a quantitative impression of standard DTI indices (FA, mean ADC, radial ADC, axial ADC), we computed averages for the sub-bundles defined regions. The axial ADC and FA may be interpreted as measures for axonal integrity. High radial ADC values may indicate an enlargement of the extra-axonal space. Group averages are shown in Table 1. Additional two DMI (Diffusion Microstructure Imaging) indices, the intra-axonal and extra-axonal volume fractions, are also reported. In DMI, the tissue is described by multiple compartments: an intra-axonal, an extra-axonal compartment and a free water compartment. While the internal diffusion parameters of the individual compartments are difficult to be determined, their relative fractions are robust parameters. For estimation, we used a previously described machine learning approach (Reisert et al., 2017).

### 2.9 Qualitative comparison of global and local tractography approaches

Fiber tractography of the slMFB was done additionally **in** our MNI group space HCP template (cf. 2.4, 2.5) based on two local approaches and compared to global tractography. For global tractography we followed (Reisert et al., 2011) as described above. For walker based tractography, a simple tensor-based FACT (https://www.uniklinik-freiburg.de/mr-en/research-groups/diffperf/fibertools.html) algorithm was used for comparison and a CSD based probabilistic tractography was performed (https://www.uniklinik-freiburg.de/mr-en/research-groups/diffperf/fibertools.html). For slMFB selection criteria cf. Section 2.6. Furthermore, a purely qualitative comparison of these three tractography approaches was performed (cf. Fig. 7) in order to investigate the effect of using local, walker based, approaches versus global approaches on the anatomical depiction of the slMFB when using a similar seeding strategy. This appears to be especially important when regarding the deterministic tracking as being the preferred “surgical” approach for targeting the slMFB (Schlaepfer et al., 2013) and other targets (Riva-Posse et al., 2017) in DBS surgery. Further detailed or quantitative comparisons between the different tracking approaches would be interesting but was beyond the scope of this work.

## 3 Results

**Fig. 2:**
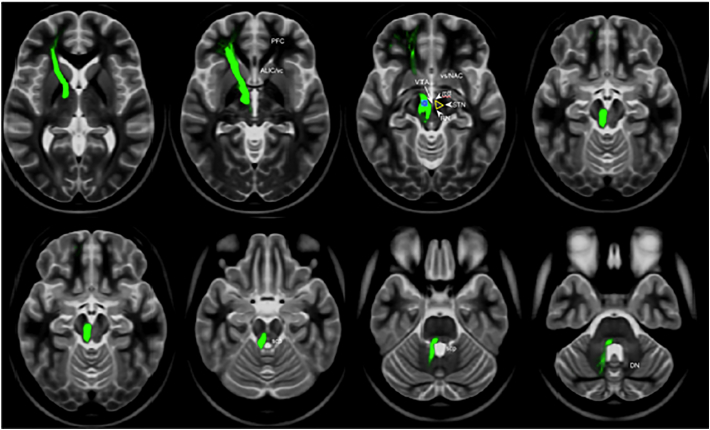
Prototypical tracking result of the MFB in MNI group space. Global tracking performed on the HCP group level. Typical seed region (blue) selected in the triangle region (yellow, contralateral) between mtt, STN and RN (lateral VTA). (Legend: ALIC, anterior limb of internal capsule; DN, dentate nucleus; mtt, mammillo-thalamic tract; PFC, prefrontal cortex; RN, red nucleus; scp, superior thalamic peduncle; STN, subthalamic nucleus; vc, ventral capsule; VTA, ventral tegmental area).

With the automated and objective analysis, the medial forebrain bundle was visualized in all subjects. slMFB - tracking in the MNI diffusion group space – which was performed to visualize an individual subject's MFB - but taking group DTI information from the HCP data into account - was achieved (Fig. 2) and simply served as an example that “prototypically” shows the MFB basic structure together with the idealized seeding region.

**Fig. 3:**
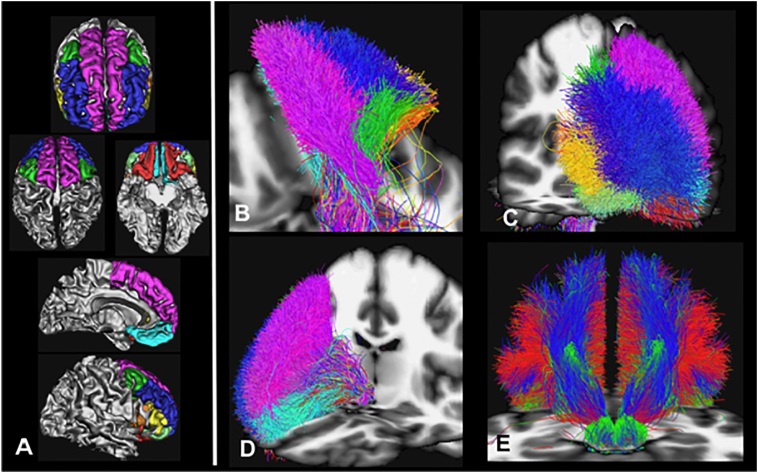
Results of global tracking (B–E) and segmentation regarding cortical parceling masks (A). B, view from dorsal; C, anterolateral right; D, antero-medial right. Fiber colors, segments: Pink, superior frontal; blue, rostral middle frontal; green, pars caudalis (of middle frontal gyrus); turquoise, medial orbitofrontal; yellow, pars triangularis; light green, pars orbitalis; not shown, pars opercularis and lateral orbitofrontal. E, view from posterior with directional color coding (blue superior/inferior, green anterior/posterior, red medial/latera). (For interpretation of the references to color in this figure legend, the reader is referred to the web version of this article.)

Fig. 3 shows an illustration of the decomposition of the MFB regarding the Desikan/Killiany cortical parceling (Fig. 3A) as target region, applied to subcortical white matter (Fig. 3B–D). Fig. 3E shows fiber directionality. The slMFB projects dominantly in three subdomains to the PFC: superior frontal, rostral middle frontal and lateral orbitofrontal.

**Fig. 4:**
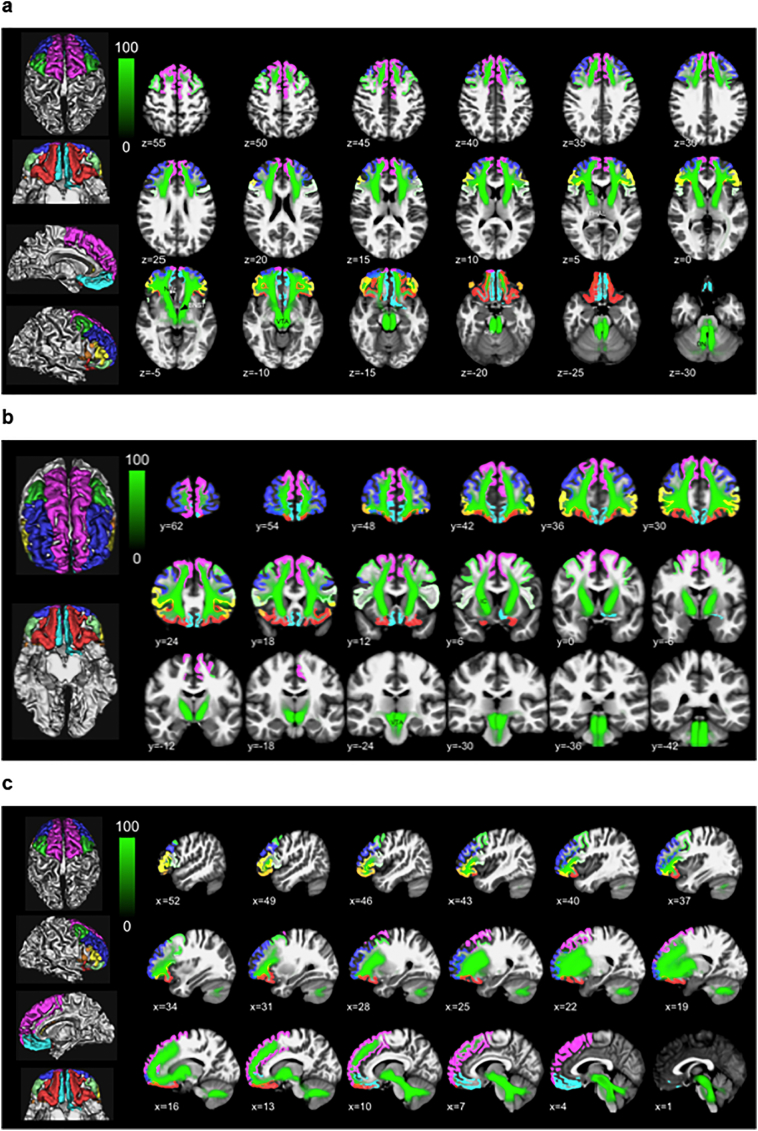
Entire MFB (green) including main trunk in MNI152 space (a, axial; b, coronal; c, sagittal). Cortical parcellation masks (left panel) in juxtaposition. Color coding in green indicates probability of occurrence of fiber-streamlines in the entire group (in percent). (For interpretation of the references to color in this figure legend, the reader is referred to the web version of this article.)

Fig. 4(a–c) shows an overview of the entire MFB structure derived by global tracking in a probability map over all subjects in MNI space with color-coded parcells overlaid on a T1w template (a axial, b coronal, c sagittal).

**Fig. 5:**
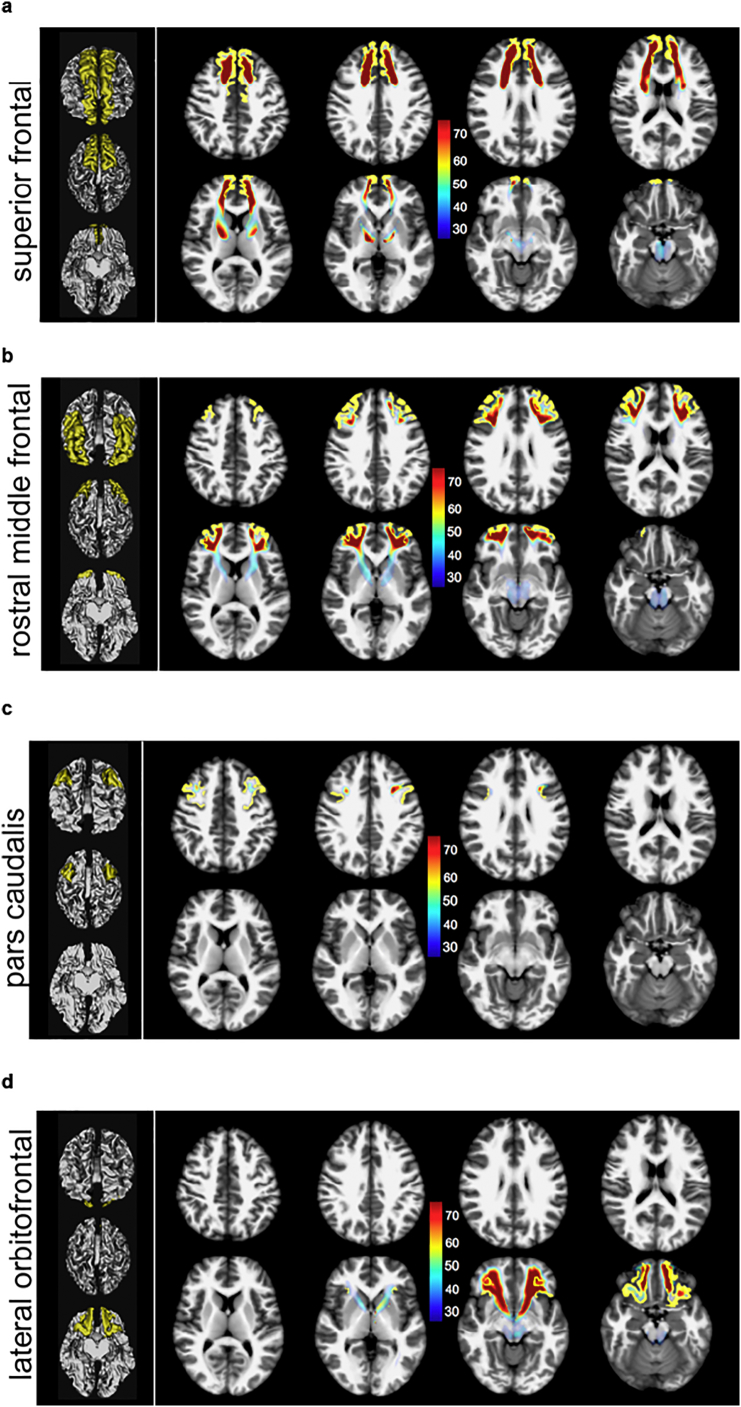

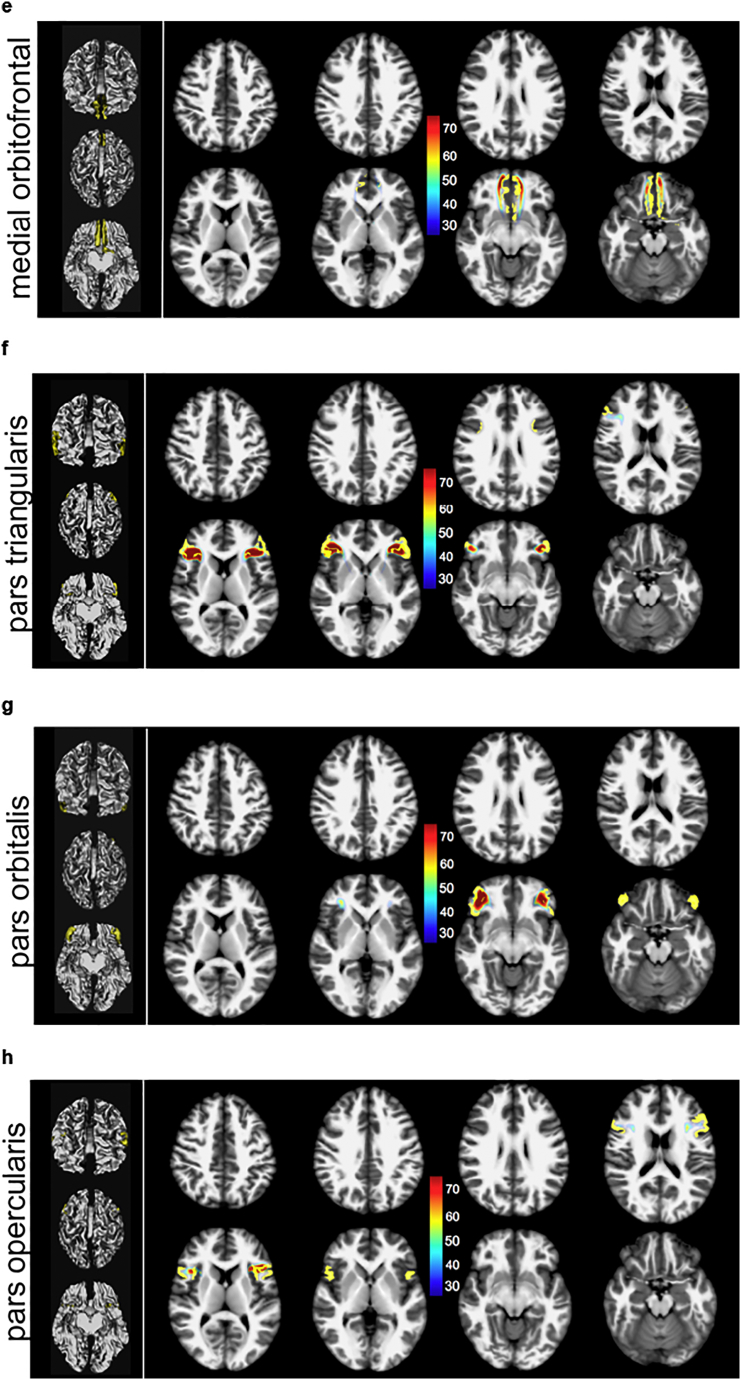
(a–h): slMFB and its prefrontal white matter (WM) sub-segments addressing distinct cortical parcellations of the PFC parceling according to Desikan/Killiany (Desikan et al., 2006) (axial slices, MNI space). Color scale shows probability of fiber occurrence in [%] relative to entire MFB structure. Global tractographic approach used. Due to the dominating nature of the superior frontal, rostral middle and lateral orbitofrontal segmentations, in these the trunk region of MFB shows up with roughly 30%.

**Fig. 6:**
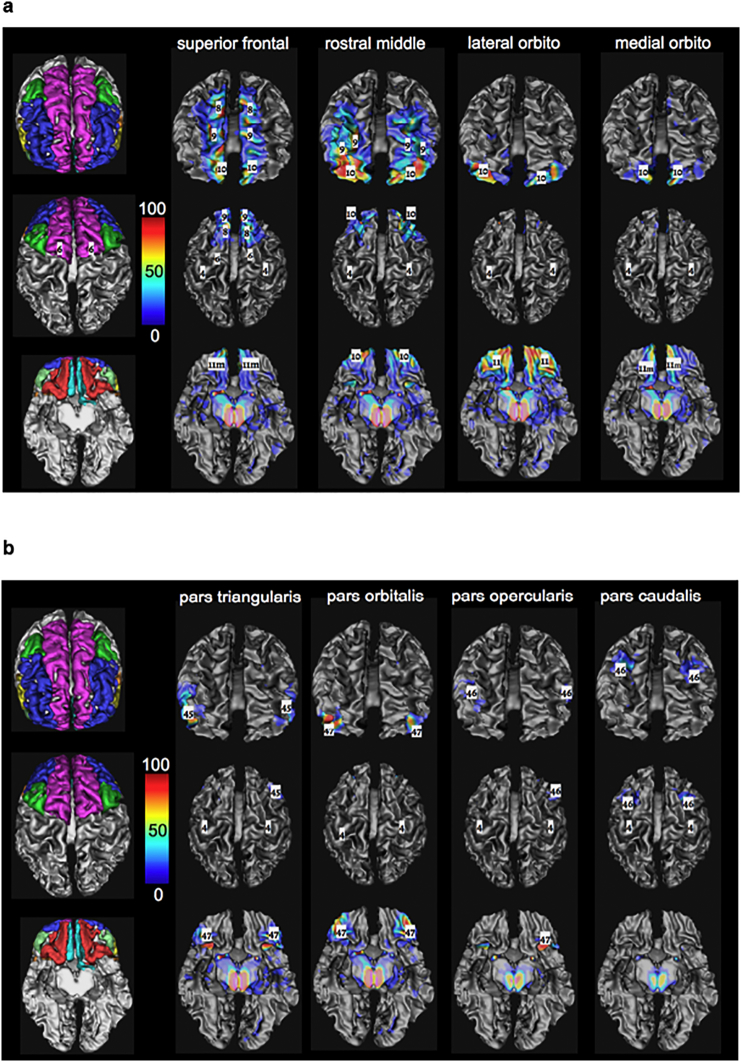
(a, b): Three-dimensional white matter of MNI. slMFB and its prefrontal sub-segments addressing distinct (WM) of overlaying cortical Desikan/Killiany parcellation in the global tractographic approach. Numbers indicate Brodmann areae (Brodmann, 1909) (left panel prefrontal parcellations, 3D representation in MNI space). Color coding represents percentage of occurrence of fibers (cf. Fig. 5) relative to the entire group.

Decompositions of the slMFB are visualized as overlay in axial T1W MNI for the individual sub-segments in Fig. 5(a–h), including color-coding (%) of the occurrence of MFB fibers. In Fig. 6 the group averages are shown as surface colorings of a white matter mask (thresholded at 0.7).

Main regions which are addressed are Brodmann areas (Brodmann, 1909) 10, 9, 8, 11, 11m. The projections to these regions contain 97% (right) and 98% (left) of the total relative fiber counts of the MFB (cf. Table 1). The superior frontal region receives fibers addressing subcortical WM and Brodmann areae (Brodmann, 1909) surrounding the anterior midline (BA 8, 9, 10); rostral middle frontal, addresses dorsolateral prefrontal cortex (DLPFC, BA 9, 10); Pars caudalis (of the middle frontal gyrus) mainly addresses BA46. Lateral orbitofrontal and medial orbitofrontal represent the OFC part of the MFB (BA 11, 11m, 10, partly BA 47); the lateral orbitofrontal parcellation shows a clear connection to the nucleus accumbens septi (NAC) and ventral striatum as subcortical reward associated structures. To reach the latter structures it passes the anterior limb of the internal capsule (ALIC). These connections are more pronounced on the left. Pars triangularis (of inferior frontal gyrus), addresses BA 45 predominantly but also WM of BA 47 (cf. Fig. 5, Fig. 6). Pars orbitalis and pars opercularis (of inferior frontal gyrus) address BA 47 and BA44, respectively.

All fibers which reach cortical regions need to pass the MFB trunk region (light blue). Table 1 further summarizes the results of percentage of MFB fibers that address the distinct regions. The indices are rather stable across the regions and agree with the usually obtained ranges.

**Fig. 7:**
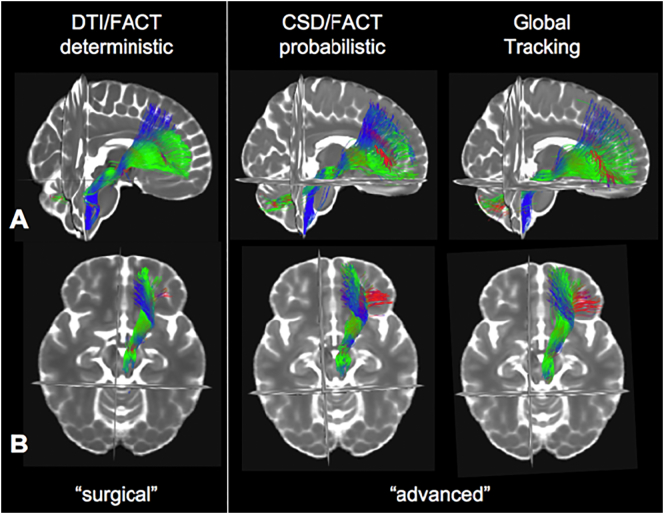
Different tracking methods applied on the group level HCP data in MNI space. The MFB main trunk is visible in all three modalities (A, sagittal; B, axial). As expected, with more advanced visualization methods (CSD/FACT probabilistic, Global) more distal and especially lateral extensions of the MFB (red) become visible (middle and right).

Fig. 7 shows the effect of the application of different tracking algorithms. The trunk region is shown in very similar extension in comparison between the distinct algorithms. Rostro-caudaul and medio-lateral fiber extensions become more pronounced in more advanced tracking (global > probabilistc tracking > deterministic).

## 4 Discussion

The MFB is an important subcortical structure of reward and motivation with far reaching cortical projections. It has the function of a regulator in the reward system. With growing interest in reward-guided learning (Neubert et al., 2015; Muhle-Karbe et al., 2016; Mars et al., 2016) and as our understanding about the consequences on the possible dysfunction of reward-related structures for affective disorders grows (Russo & Nestler, 2013; Blood et al., 2010a; Schlaepfer et al., 2014; Schlaepfer et al., 2008), thorough knowledge on the anatomy of the MFB becomes mandatory.

### 4.1 MFB connections to reward-associated PFC and OFC

Recently the Desikan/Killiany automated parcellation of cortical structures was introduced which we used to objectively parcel the prefrontal cortex in our cohort (Desikan et al., 2006). Since Brodmann's definition of distinct cerebral cortical regions (Brodmann, 1909) still has some value in a neuroscientific context for description of localization of function (for an overview cf. Petrides (2005)) and in order to facilitate the discussion - especially in the field of DBS (Riva-Posse et al., 2017; Riva-Posse et al., 2014; Johansen-Berg et al., 2008) - we have in part adapted our results and compared them to the Brodmann nomenclature.

The MFB projects dominantly in three subdomains to the PFC: superior frontal, rostral middlefrontal and lateral orbitofrontal. These projections contain 97% (right) and 98% (left) of the total relative MFB fiber count (cf. Table 1). The following discussion of the sub-parcellation especially with respect to Brodmann areas is non-exhaustive and interpreted to the relevance of the slMFB distribution with respect to affective disorders and DBS evaluation. The comparison of the subparcellation of the PFC with Brodmann regions can be seen in Fig. 6.

#### 4.1.1 Superior frontal parcellation

This parcellation addresses subcortical WM and Brodmann areas surrounding the anterior midline (BA 8, 9, 10) thus representing the medial prefrontal cortex (mPFC) (la Vega de et al., 2016). It contains 38% (right) and 31% (left) of the total MFB frontal projections (cf. Table 1). *BA10* is the largest cyto-architectonic region of the human brain and is anatomically identical with the anterior or rostral prefrontal cortex (Burgess et al., 2007). Brodmann described it as covering the frontal pole in the anterior third of the first and second frontal gyrus. On the medial surface, it does not reach the calloso-marginal sulcus. Although this region is poorly understood it appears to be important as a regulator of frontal lobe function with respect to the general working memory. According to a meta-analysis from 104 functional studies, Gilbert et al. were able to divide the anterior PFC (aPFC) into three regions (Gilbert et al., 2006). They conclude that the aPFC is functionally inhomogeneous. In the medial-lateral gradient the aPFC serves the purpose of “multitasking” (medial) and “episodic memory” (lateral). In the dorsal midline the aPFC is involved in “mentalizing”.

So far emotion regulating inputs into BA10 (and BA9) have been described entering via the medial prefrontal cortex (mPFC). Recently, de la Vega et al. (la Vega de et al., 2016) found evidence for a tripartite functional segmentation of the mPFC in a meta-analysis of nearly 10.000 fMRI studies with an anterior-dorsal gradient. The most anterior one was associated with reward, social processing and episodic memory (BA10), the dorsal one was associated with motor planning (BA6, 8) and the intercalated one was associated with pain and affect (la Vega de et al., 2016). In our results BA10 is addressed more from a lateral entry (via the *rostral middle frontal parcellation*, see below, Fig. 4, Fig. 5, Fig. 6) possibly entering BA10 more in the “episodic memory/mentalizing functional region” according to Gilbert et al. (Gilbert et al., 2006). Other groups have looked at distant connectivity of their DBS targets for major depression (vc/vs and cg 25) and found similar aspects for ventral capsule/ventral striatum - which mainly connects to the PFC via the MFB - and a more midline addressing part concerning BA10 for cg25 (Gutman et al., 2009). Obviously, these DBS target sites for major depression (vc/vs, slMFB and cg25) in principle affect different parts of the aPFC (BA10). *BA8* is a functionally small region. This region has been reported (amongst other regions) to deal with uncertainty (Yoshida & Ishii, 2006). BA8 also contains the frontal eye fields and is believed to be concerned with conjugate eye movements. Some researchers think that the region is concerned with hope (Chew & Ho, 1994). BA9 is concerned (amongst other reported functions) with auditory and verbal attention, inferring the intention of others and sustained attention.

#### 4.1.2 Rostral middle frontal parcellation

*Dorsolateral prefrontal cortex (DLPFC, BA 9, 10*), this parcellation contains 35% (right, left) of the frontally projecting VTA-PFC connection. BA9, 10 have been addressed above. BA32, is typically active during rational thought processes but has close association to BA24.

#### 4.1.3 The Lateral orbitofrontal and medial orbitofrontal

Parcellations represent the OFC part of the MFB projection (BA 11, 11m, 10, partly BA 47). Orbitofrontal cortex (BA11/11m): This region is related to reward interpretation an evaluation of importance of an object and the reward value associated with it. It deals with the value of an anticipated reward in contrast between different object of anticipation. According to other views, the mOFC (BA 11m) is related to reward, while lOFC (BA11 and BA47) are more dealing with interpretation of punishment (Kringelbach & Rolls, 2004). It can be speculated that dysfunctional BA11m (or disrupted connection between VTA and BA11m) might in certain subtypes of depression result in ruminations because of a lack of reward prediction. *BA47 itself* was initially described by Brodmann as “speech related”. It is the orbital part of the third frontal gyrus (inferior frontal gyrus). According to newer studies this region might together with BA10 and BA46 be related to the emotional reappraisal of memories especially on the right side. Today, BA47 is allocated as being functionally closer to BA11, thus the OFC, than to functional speech domains. BA47 is involved in judgment of familiarity of stimuli during task planning *(**Petrides, 2005**).*

#### 4.1.4 Lateral orbitofrontal parcellation

Subcortically shows a clear connection to the nucleus accumbens septi (NAC) and the ventral striatum which are reached via the anterior limb of the internal capsule (ALIC) (cf. Fig. 5d and 6a). This parcellation contains 24% (right) and 32% (left) of the prefrontal MFB projections. Cortically, mainly BA11, BA10 and BA47 are involved. **Pars orbitalis and *pars triangularis*** address predominantly the white matter of BA47, and BA45, respectively. **Pars caudalis (of the middle frontal gyrus)** extends toward BA 46 (bilaterally, together with BA9) which has recently been identified as being related to the planning, evaluation and executions of future motor tasks (Nitschke et al., 2016).

### 4.2 Comparative primate anatomy

With a lack of gross histological description of the human MFB which probably is related to the lack of myelin and a complicated path the structure takes through the brain, a comparison to other primate species might yield greater insight. In the macaque monkey (Frankle et al., 2006) the PFC – VTA connection appears to be only sparsely developed. On the contrary, human DTI studies (including this one) mainly show a dense connection of PFC and midbrain (including VTA). These results are substantiated by earlier degeneration work in humans in which a direct connection of the PFC to the midbrain was described (Hurwitz et al., 2006; BECK, 1950). In the macaque the PFC – midbrain connection to the subthalamic nucleus (STN) and the substantia nigra (SN) appear to consist of a denser fiber network than the connection to the VTA (Haynes & Haber, 2013). Haynes and Haber used retrograde cortical injections in the macaque monkey and find a direct connection of the medial – limbic - STN tip and the PFC. A similar pathway was already mentioned by our group in human DTI studies (Coenen et al., 2011; Coenen et al., 2009; Coenen et al., 2012a), which can be regarded as a non-invasive human albeit less specific analogue to tract-tracing studies. We described tributaries to the STN which connect the limbic STN to the MFB - and possibly the PFC - in our initial description of the human MFB. Inadvertent stimulation of these tributaries (or of the MFB main trunk in their proximity) with DBS might result in a hypomanic state in the human and have been offered as an alternative explanation for this pathology as opposed to pure medial limbic STN stimulation (Coenen et al., 2009). Nieuwenhuys has located the MFB in the exact same place, just lateral to the red nucleus and medial to the STN in his work (Nieuwenhuys et al., 2008). This description, however, is vague with respect to more distal extensions and also represents an extrapolation from animal work. Cross-species comparative results need to be interpreted cautiously, since the dense human slMFB might have developed as a major connection pathway between VTA and the evolutionary enlarged anterior prefrontal cortex (especially BA10, see above). The latter structure is less developed in non-human primates with significantly larger volume in the human (Burgess et al., 2007). Probably a smaller PFC with its distinct anatomical features explains a rather sparse connection of PFC and midbrain VTA in the macaque. In the light of this analysis, the macaque hyperdirect “limbic” PFC-STN pathway (Haynes & Haber, 2013) is the primate correlate to the earlier described human slMFB (Coenen et al., 2009; Coenen et al., 2012a).

### 4.3 Choice of the tractographic method to visualize the MFB with respect to surgical planning for DBS

In recent years DTI fiber tractography has become an important tool to plan surgical interventions and stimulation with DBS (Calabrese, 2016; Coenen et al., 2012b; Coenen et al., 2017). In fact, all licensed stereotactic software solutions that allow fiber tractographic planning are based on a deterministic single tensor approach typically using FACT (fiber assignment by continuous tracking) or HAFT (High Angular Fiber Tracking) algorithms. The slMFB DBS target for depression was the first true fiber-tractographic target site for DBS (Coenen et al., 2011; Schlaepfer et al., 2013). Meanwhile other groups have followed this approach and introduced fiber tractography to improve their results in DBS for depression (Riva-Posse et al., 2017; Riva-Posse et al., 2014) while so far relying on a gross anatomical approach (Hamani et al., 2009). They now use clinical DWI datasets to identify a template that shows the rough and deterministic information of four crossing pathways needed for successful stimulation of the cg25 region. They cross check with an MNI template that is determined with advanced tractography methods. We have here compared different tractographic approaches for the slMFB-DBS target (cf. Fig. 7). This comparison was simply intended to show, that a single tensor – yet clinically applicable and standard – deterministic approach (including DTI) will not be sufficient and prove inferior to advanced visualization strategies (including obtaining directional information with CSD and not DTI) to show the distal branching into distinct segments especially with the slMFBs medial-lateral and rostro-caudal gradients. There are obviously other tractographic methods available. However, targeting with deterministic methods for the main trunk of the MFB appears to be feasible, since in this part there are no conflicts with crossing, branching and kissing fibers that might introduce errors in deterministic tractographic rendition. However, this is not the focus of this research.

### 4.4 Significance with respect to affective disorders

The described work might help to understand key symptoms of depression and conceptualize them with a loss of connectivity to certain PFC structures (OFC = reward attribution, BA47 = emotion reappraisal, BA10/DLPFC = goal directed behaviors). Obviously, there have already been authors who have speculated about the reward system's role in depression genesis (Russo & Nestler, 2013; Coenen et al., 2011; Bracht et al., 2014a; Bracht et al., 2014b; Schlaepfer et al., 2014; Schlaepfer et al., 2008; Blood et al., 2010b).

### 4.5 Limitations

We have here exclusively looked at connections between VTA and the PFC/OFC regions including some structures of passage (ALIC, NAC). Specific connections amongst these regions themselves were not part of this analysis and our work was not designed to look at the organization between PFC/OFC sub-regions. The MFB sub-segmentation as described here (and according to cortical parceling) is artificial and different from previous sub-segmentation (Zacharopoulos et al., 2016; Bracht et al., 2014a; Bracht et al., 2014b; Bracht et al., 2015). It is therefore not possible to directly compare microstructural DTI/DMI values with previous work. Moreover, each MFB segment has to pass the “bottle neck” of the main MFB trunk (close to the lateral VTA, cf. Fig. 5a, b, d) which will heavily influence measurements of microstructural features of each segment.

While fiber tractography can delineate the MFB (better the slMFB) on high quality template data consistently (like in individual HCP tracking, cf. Fig. 2), tractography in typical subject space is still challenging and highly dependent on details of the acquisition scheme, preprocessing steps and tracking methods. In this respect, anisotropic voxel acquisition - like we have performed here – might be regarded as a limitation (Campbell & Pike, 2014). In particular, for more sophisticated tracking algorithms that can also cope with crossing situations, the choice of the algorithm and its parameters has a high impact on the results, mainly caused by the inherent shortcomings of diffusion weighted imaging, like the kissing/crossing problem, noise, low resolution and image distortions. Also, brain morphology with its impact on fiber geometry has to be taken into account as a confounding factor, and has to be separated from relevant changes of diffusion properties in myelinated white matter (Campbell & Pike, 2014). However, the shown agreement of the bundle structure, even with simple deterministic DTI based algorithms (cf. Fig. 7), gives strong evidence for the existence and robustness of the herein and previously described (Coenen et al., 2009; Coenen et al., 2012a) MFB.

## 5 Conclusion

Far reaching reward associated connections of the VTA with prefrontal regions exist in human. These connections can only partially be found in non-human primates. The MFB system serves as a connection hub between subcortical evolutionary ancient (e.g. VTA) and evolutionary more recent - cortical – structures. More detailed knowledge about the connections of the structures of reward and motivation might help to develop more personalized interventions directed to prefrontal structures like TMS or continuous cortical stimulation in major depression (Pathak et al., 2013) - whose key symptoms are hopelessness and anhedonia - and other neuropsychiatric disorders. DBS of the slMFB is currently opening up as a valid therapeutic avenue for depression and also other DBS interventions (targeting cg 25 and vc/vs) show their influence on the PFC/OFC. In future work, therapeutic effects of any of these interventions can be evaluated by 1) the mere count of fibers addressed (using electric field modelling techniques), 2) the distinct reward and learning associated regions involved, 3) connectivity between these regions and 4) pre- and post-interventional microstructural changes. A thorough anatomical and microstructural description of the reward related PFC/OFC regions and their connection to the VTA - the MFB - is mandatory in order to enable an interpretation of distinct therapeutic effects from different interventional treatment modalities in neuropsychiatric disorders. Our detailed normative map of the MFB derived from an advanced analysis tool might - as an anatomical template – in the future guide the less detailed deterministic tractography efforts with standard planning software (Schlaepfer et al., 2013; Coenen et al., 2017) to target for DBS in depression and other disorders related to dysfunction of reward and reward-associated learning.

## Disclosures

**Unrelated:** VA Coenen has received honoraries and travel expenses for talks from Medtronic (USA), Boston Scientific (USA), Desitin (Europe). He has received limited funding for (in part ongoing) IITs from Boston Scientific and Medtronic and for collaborative work with Brainlab AG (Feldkirchen, Germany). VA Coenen is member of the scientific advisory board for CorTec (Freiburg, Germany). M Reisert was funded by grant from BrainLab AG (Feldkirchen, Germany).

## Aknowledgements

**HCP acknoledgement:** Data used in the preparation of this work were obtained from the MGH-USC Human Connectome Project (HCP) database (https://ida.loni.usc.edu/login.jsp). The HCP project (Principal Investigators: Bruce Rosen, M.D., Ph.D., Martinos Center at Massachusetts General Hospital; Arthur W. Toga, Ph.D., University of California, Los Angeles, Van J. Weeden, MD, Martinos Center at Massachusetts General Hospital) is supported by the National Institute of Dental and Craniofacial Research (NIDCR), the National Institute of Mental Health (NIMH) and the National Institute of Neurological Disorders and Stroke (NINDS). Collectively, the HCP is the result of efforts of co-investigators from the University of California, Los Angeles, Martinos Center for Biomedical Imaging at Massachusetts General Hospital (MGH), Washington University, and the University of Minnesota.
